# Young people's perspectives on addressing UK health inequalities: utopian visions and preferences for action

**DOI:** 10.1111/hex.13825

**Published:** 2023-07-24

**Authors:** Gillian Fergie, Caroline Vaczy, Katherine Smith, Mhairi Mackenzie, Thu Thuy Phan, Shona Hilton

**Affiliations:** ^1^ MRC/CSO Social and Public Health Sciences Unit School of Health and Wellbeing, University of Glasgow Glasgow UK; ^2^ Centre for Health Policy School of Social Work and Social Policy, University of Strathclyde Glasgow UK; ^3^ School of Social and Political Science University of Edinburgh Edinburgh UK; ^4^ Urban Studies School of Social and Political Sciences, University of Glasgow Glasgow UK

**Keywords:** arts‐based research, health inequalities, lay perspectives, public policy, United Kingdom, young people

## Abstract

**Introduction:**

It is increasingly recognised by UK researchers and population health advocates that an important impetus to effective policy action to address health inequalities is activation of public dialogue about the social determinants of health and how inequalities might be addressed. The limited body of existing scholarship reaches varying conclusions on public preferences for responding to health inequalities but with consensus around the importance of tackling poverty. Young people's perspectives remain underexplored despite their increasingly visible role in activism across a range of policy issues and the potential impact of widening inequalities on their generation's health and wellbeing.

**Methods:**

Six groups of young people (39 in total) from two UK cities (Glasgow and Leeds) were engaged in online workshops to explore views on health inequalities and potential solutions. Inspired by calls to employ notions of utopia, artist–facilitators and researchers supported participants to explore the evidence, debating solutions and imagining a more desirable society, using visual and performance art. Drawing together data from discussions and creative outputs, we analysed participants' perspectives on addressing health inequalities across four domains: governance, environment, society/culture and economy.

**Findings:**

Proposals ranged from radical, whole‐systems change to support for policies currently being considered by governments across the United Kingdom. The consensus was built around embracing more participatory, collaborative governance; prioritising sustainability and access to greenspace; promoting inclusivity and eliminating discrimination and improving the circumstances of those on the lowest incomes. Levels of acceptable income inequality, and how best to address income inequality were more contested. Individual‐level interventions were rarely presented as viable options for addressing the social inequalities from which health differences emanate.

**Conclusion:**

Young people contributed wide‐ranging and visionary solutions to debates around addressing the enduring existence of health inequalities in the United Kingdom. Their reflections signal support for ‘upstream’ systemic change to achieve reductions in social inequalities and the health differences that flow from these.

**Public Contribution:**

An advisory group of young people informed the development of project plans. Participants shaped the direction of the project in terms of substantive focus and were responsible for the generation of creative project outputs aimed at influencing policymakers.

## 1 INTRODUCTION

The social and structural causes of inequalities in population health are well established.^1^ For those committed to reducing health inequalities, however, there has been a frustrating lack of progress,^2^ which has stimulated calls for wider public conversations on the social determinants of health to better support effective policy action.^3^, ^4^ Some qualitative studies have explored UK public perspectives on health inequalities but few explore public views of potential policy responses.^5^ This is an important gap since lack of public support is often cited as a barrier to implementing evidence‐informed policy responses to health inequalities.^6^, ^7^ The studies that do exist, two of which employed Q‐methodology^8^, ^9^ and one which combined citizens' juries and a national survey,^10^ all found the public willing and able to engage in discussions.^8^, ^9^, ^10^ However, perhaps unsurprisingly given the range of communities and methods involved, there are no consistent preferred solutions across these studies. There does, however, appear to be some consensus across studies that poverty and public services require greater policy attention.^8^, ^9^, ^10^ Smith et al. found that public support was greatest for proposals to reduce health inequalities via improvements to living and working conditions and more redistributive economic policies, and noted that support for these kinds of ‘upstream’ policies increased when participants worked collectively in citizens' juries.^10^ Using Q‐methodology, McHugh et al. found agreement, between professional stakeholders and community members, around the importance of ensuring that people have enough money to meet basic needs and support for mitigating the negative impacts of unpredictable finances, job insecurity and welfare benefit cuts.^8^ None of these studies had a specific focus on young people despite the importance of structural factors to young people's health.^11^

The limited research specifically exploring views of young people on health inequalities suggests they articulate well socioeconomic drivers of population‐level health inequalities, notably income and employment.^12^ Children's accounts stand out from adult perspectives^5^ in also emphasising education and relationships,^13^ perhaps reflecting concerns of particular importance to their life stage.^13^ Views on possible solutions, however, have largely been neglected. Often described as a key group with whom political trust needs to be built,^14^ young people are potentially key stakeholders in policy decision‐making to address health inequalities. The disproportionate impacts on this group of rapid changes in employment opportunities and labour markets,^15^, ^16^ disruption to education and educational transitions^17^, ^18^ and trends in worsening mental health^19^ have led to representations of Gen Z (those born between the mid‐1990s and early 2010s) as a ‘lost’^20^ or ‘precariat’^21^ generation, experiencing considerable intergenerational inequalities. Given this burden and the key role of youth activism in demanding policy change across a range of policy domains,^22^ young people's perspectives on how health inequalities might be addressed seem a critical part of public conversations and advocacy efforts.

Scott‐Samuel and colleagues propose that research exploring potential responses to health inequalities might be aided by engagement with notions of utopia.^23^, ^24^ By working with communities, including those most negatively impacted by health inequalities, to ‘sketch out collaborative ideas for how our society might be organised and governed’, they suggest new imaginative solutions might be envisaged to progress policy action.^24^,^p.429^ Levitas^23^,^p.153^ describes imagining utopia as ‘speculative sociology’, using the reality of the present as a foil against which possibilities for a better world can be imagined via individual and collective processes. In relation to addressing health inequalities, these processes could precede the generation of consensus around actionable solutions. In this study, we asked young people about their perceptions of health inequalities and potential alternative visions for fairer societies in which health inequalities are reduced. Specifically, the research presented here focuses on the following exploratory questions: 1—What do young people, drawn from across Glasgow and Leeds (United Kingdom), envisage a fairer society, in which health inequalities are reduced, would look like? 2—What are young people's perceptions of key policy domains for addressing inequalities and what actions do they support in reducing inequalities?

## 2 METHODS

### 2.1 Methodological approach, setting and partnerships

By employing arts‐based research with young people, we aimed to provide safe and collaborative spaces (with artists and researchers) to discuss how health inequalities are generated and might be addressed. Developed largely in the tradition of participatory research, arts‐based approaches have been adopted across disciplines, including health,^25^ generally engaging underserved populations, including young people.^26^ Glasgow and Leeds were identified as study sites because of their similarities, as important regional urban centres in the United Kingdom, home to large and diverse populations and key differences in the political contexts and governance arrangements. Reducing health inequalities is an ongoing focus of local government and public health bodies in both cities.^27^, ^28^ We partnered with creative organisations with an interest in supporting creative community engagement in each city, Impact Arts (Glasgow); Leeds Playhouse and Opera North (Leeds). The project design was co‐produced with artist–facilitators from each organisation, building upon their expertise in creative capacity building, online facilitation and safeguarding and local knowledge.

### 2.2 Sampling and recruitment

Six groups of young people, three in Leeds and three in Glasgow, were recruited to participate in arts‐based workshops over 4 days (see Table 1). Groups of young people (aged 14–20) who had a connection with our partner organisations were contacted by email or WhatsApp with project details. Participants were likely to have a pre‐existing interest in the arts, and had been involved in youth groups or activities with various social aims: addressing youth mental health, women's empowerment, improving employability or supporting community cohesion. Those interested contacted creative facilitators for information, which was then provided in text and video format. Participants were given art materials, a tote bag and £100 as compensation for their time. Thirty‐nine young people participated.

**Table 1:** Group details.

City (group identifier)	Group origins	Sample size	Ages	Preferred pronouns		
				She/her	He/him	They/them
Glasgow 1	Community programme focused on creativity for young people who want to build confidence	9	14–17	4	5	0
Leeds 1	Community outreach project within a diverse neighbourhood with a rich social history of immigration	6	14–20	5	1	0
Leeds 2	Alternative education programme delivered through creative practice	5	17–20	3	2	0
Glasgow 2	Various youth arts programmes focused on confidence‐building or employability	5	14–19	3	1	1
Leeds 3	Programmes/organisations focused on empowering young women and supporting youth mental health	7	14–17	6	0	1
Glasgow 3	Employability programme focused on creativity	7	16–19	5	2	0
Totals	Glasgow: *n* = 21		14, *n* = 4	26	11	2
	Leeds: *n* = 18		15, *n* = 6			
	Overall: *n* = 39		16, *n* = 6			
			17, *n* = 10			
			18, *n* = 4			
			19, *n* = 7			
			20, *n* = 2			

### 2.3 Workshops

Although in‐person workshops were planned, data collection (October 2020 to May 2021) was impacted by pandemic‐related physical distancing measures in the United Kingdom, so workshops were online using the preferred video‐conferencing platforms of partner organisations. This pivot involved close consideration of the potential for digital exclusion and accessibility needs of participants, development of alternative engagement and facilitation approaches and rapid innovation in collaborative online art‐making activities. Materials (including art supplies, project information, public health evidence, weekly schedules and laptops, if required) were posted to participants before the first workshop. Each day comprised three/four workshop sessions, with breaks between. Young people joined from private spaces, mostly their homes. Workshops were led by creative facilitators, drawing on activities, resources and topic guides co‐developed with researchers. Facilitators completed a short online training module in health inequalities as preparation for the project but had not previously engaged with evidence or policy discussion in the area. Their expertise was in creative engagement and facilitation with young people, and their role in the workshops was collaborative and supportive, rather than primarily focused on data collection. Researchers observed sessions, answered questions and offered facilitation support as needed.

Following the first workshop series (Glasgow 1), session plans were refined based on facilitator, researcher and participant insights. In the five subsequent workshops, the first day was used to explore drivers of health inequalities while the remaining days focused on addressing inequalities. An indicative schedule for workshops is provided in Table 2.

**Table 2:** Indicative workshop schedule.

Day 1	Day 2	Day 3	Day 4
Introductions	Warm‐up	Warm‐up	Warm‐up
1. Facilitator overview of the week 2. Introductions to each other 3. Group discussion: Code of conduct discussion	1. Facilitator welcome 2. Warm‐up activities Performance art: Mime of repetitive movements of lockdown Visual art: Single‐line drawing of something important to me today	1. Facilitator welcome 2. Warm‐up activities Performance art: Mime of repetitive movements of lockdown Visual art: Single‐line drawing of something important to me today	1. Facilitator welcome 2. Warm‐up activities 3. Reflections on priorities and how these may have changed over the course of the week
**Break**	**Break**	**Break**	**Break**
Influences on health	Policy preferences for action on influences on health	Imagining Utopia: A better world with reduced health inequalities	Showcase: Sharing creative pieces
1. Group discussion: What makes a healthy person? 2. Kahoot quiz: Exploring evidence on health inequalities 3. Group discussion and ranking activity: What are the most important influences on health?	1. Evidence sharing: Range of articles and videos on the pandemic, young people and inequalities 2. Policy polls on Zoom (for a range of policy domains discussed on Day 1 (e.g., housing, income, education) with options to choose individual‐level, targeted and universal policy actions 3. Group discussion on preferences, reasoning and understanding of implications	1. Facilitator introduction to the concept of utopia, shares examples from popular culture and poses the question what would a better world/society without health inequalities be like? 2. Individual visions—creating lists, maps and images of utopia 3. Group discussion: Features of our utopias and policy action needed 4. Small group discussions—translating calls for action to artwork	1. Showcase planning and run through/rehearsal 2. Showcase with an invited audience 3. Reflections from audience 4. Participant postshowcase check‐in
**Break**	**Break**	**Break**	**Break**
Creative activities	Creative activities	Creative activities	Group check‐in/cool down
Art‐making responsive to the COVID‐19 pandemic	Art‐making responsive to potential policy actions on health inequalities	Final art‐making summarising calls for action on health inequalities	Group discussion: Evaluation
Performance art: Creative writing about experiences of the pandemic	Performance art: Creative speech writing focused on advocating for change to address inequalities	Performance art: Spoken word performances developed	Final check‐in/cool down
Visual art: Pandemic dialectogram—drawing maps that depict places and how they are used.	Visual art: Creating slogans and logos through printmaking techniques, building printing capacity	Visual art: Final printing session, feedback from facilitators, refining prints	Thanks and final reflections from participants, facilitators and researchers
	1:1 interviews conducted simultaneously	1:1 interviews conducted simultaneously	
Group check‐in/cool down	Group check‐in/cool down	Group check‐in/cool down	
Sharing work‐in progress and group discussion	Sharing work‐in progress and group discussion	Sharing final pieces and group discussion	

Sessions included warm‐up games; group discussions, breakout discussions and interviews; engagement with research evidence and policy and responsive creative practice. Glasgow groups focused on visual arts and Leeds groups focused on performance arts according to the expertise of facilitators. Young people were supported to develop creative outputs through engagement with notions of utopia and alternative futures,^24^ producing prints or posters (in Glasgow) and performance art (in Leeds) that reflected their understandings of alternative better futures or the policy action needed to move towards these visions. The workshop content was also driven by each group's interests, with young people creating artwork that reflected their priorities. Workshops culminated in a showcase webinar in which participants shared their work with invited artists, researchers, friends and community/youth engagement professionals. Selected artworks and extracts from performances were included in a Zine and short film available online.^29^ All sessions were recorded, with participants' consent. Ethical approval was granted by the University of Glasgow College of Social Sciences Research Ethics Committee, application number 400200006. Sessions generally kept to time but were facilitated in a relatively informal way by creative facilitators to create a supportive and friendly environment, in which participants could contribute freely.

### 2.4 Analysis

The creative activities, group discussions and interviews resulted in a diverse data set, including verbatim transcripts derived from recordings of 51 group and breakout discussions and 32 interviews, ethnographic notes, extracts of creative writing and visual and performance art (dialectograms, photographs, posters and videos). Text files were uploaded to NVivo 12 for coding. Lead and second authors read and reread all materials and developed an initial thematic network summarising visually the main themes and sub‐themes, and relationships between these.^30^ These initial themes and subthemes reflected participants' emergent priorities and concerns. By cross‐referencing these with Whitehead's typology of actions to tackle social inequalities in health,^31^ we constructed an abductive framework^32^ featuring categories of visions and policy actions. The framework was discussed and refined before being applied to all files by the second author. Specific policy proposals developed by young people were also considered in relation to policy recommendations from wider population health research. Artworks were grouped according to the thematic framework developed and analysed alongside transcripts. This provided an opportunity for triangulation, as described in other qualitative research using artwork as data,^33^ across individual accounts, group discussions and artworks, to confirm interpretations.

## 3 FINDINGS

Our analysis of young people's discussions and creative outputs shows engagement across wide‐ranging policy domains influencing health inequalities. We briefly discuss participants' conceptions of effective solutions, before considering their responses to the task of envisaging what a society in which health inequalities are much reduced might look like.

### 3.1 Defining problems and effective solutions

Within and across groups, there was agreement on the need to address health inequalities, and broadly groups appeared to conceptualise health as socially determined. This may reflect, in part, responses to evidence participants encountered early in the workshops describing the nature and extent of the problem.^3^, ^20^ However, these framings were supported by participants' own nuanced articulations of the drivers of health inequalities, and evident too in their preferences for solutions. For example, in responding to potential policy options for improving mental health and addressing inequalities, one participant commented:

G2_P3: I knew that the mental health services in school wouldn't help at all, that the schools just don't listen, even though they said they did, but they just don't. And the [free Mindfulness] app would just be easily forgotten, just like if you were to download a game, play it for a bit, use it, then just forget about it and delete it. And, just, so protecting people's jobs relieves stress and just gives them a proper income, so they have money to live and have a house, the standard things that they need for living. (Glasgow 2, she/her, 15)

Across discussions, participants' preferred actions to tackle health inequalities could be considered ‘upstream’ policy changes. Efforts to secure adequate living and working conditions for all, through systemic change, were frequently described as more likely to impact health inequalities than individually focused interventions. Indeed, young people's proposals align with expert characterisations of effective interventions as addressing causes rather than symptoms of health inequalities^31^ and echo wider (adult) public perspectives.^10^

### 3.2 Imagining alternative futures

Young people shared their creative visions for alternative futures in which health inequalities are reduced and developed a series of ‘policy asks’ for addressing social inequalities. Explanation of the mechanisms and pathways through which these linked to health varied across policy domains discussed. Similarly, progression from visions to policy asks varied: some participants stayed committed to alternative future visions throughout discussions, even if far removed from reality, while others progressed from utopian visions to proposals for refining current systems and policies as incremental steps towards a longer‐term vision.

Our analysis categorised participants' reflections into four overlapping domains: governance; environment; society/culture and economy. We describe each of these in turn, providing illustrative examples of the visions described, noting underpinning values and proposals for action and considering consensus and diversity.

#### 3.2.1 Participatory future—governance

Many participants shared visions of alternative futures that involved replacing or enhancing systems of governance to address inequalities in power. Improved systems were associated with cooperation, transparency and devolution.

Facilitator: [L3_P2], in your utopia, what does power and responsibility look like?L3_P2: […] there wouldn't be a government, it would be sort of socialist, but not like…because there's, like, Communism, where everyone is the same, and you do everything the same. But it would be like, everyone works together, [rather] than have discrimination, or just like, splitting people apart, it would be a communion. (Leeds 3, she/her, 16)

This participant shared concerns surrounding historic examples of Communism and infringement of freedoms, alongside a desire for a shift from the current system to one centred around community and cooperation. Similarly, more community‐based governance was proposed by another participant:

L3_P4: […] the community would run the community. So it would just be normal people […] And you can go to, like, the big town hall, or something, have a discussion, get to know each other's points, have a bit of an argument and debate […]. Because there's nothing saying that we can't do the job that they're [current UK government] doing, […] if we managed to get our idea out there, into our own communities, we could be the change that we need. (Leeds 3, she/her, 16)

**Figure 1:**
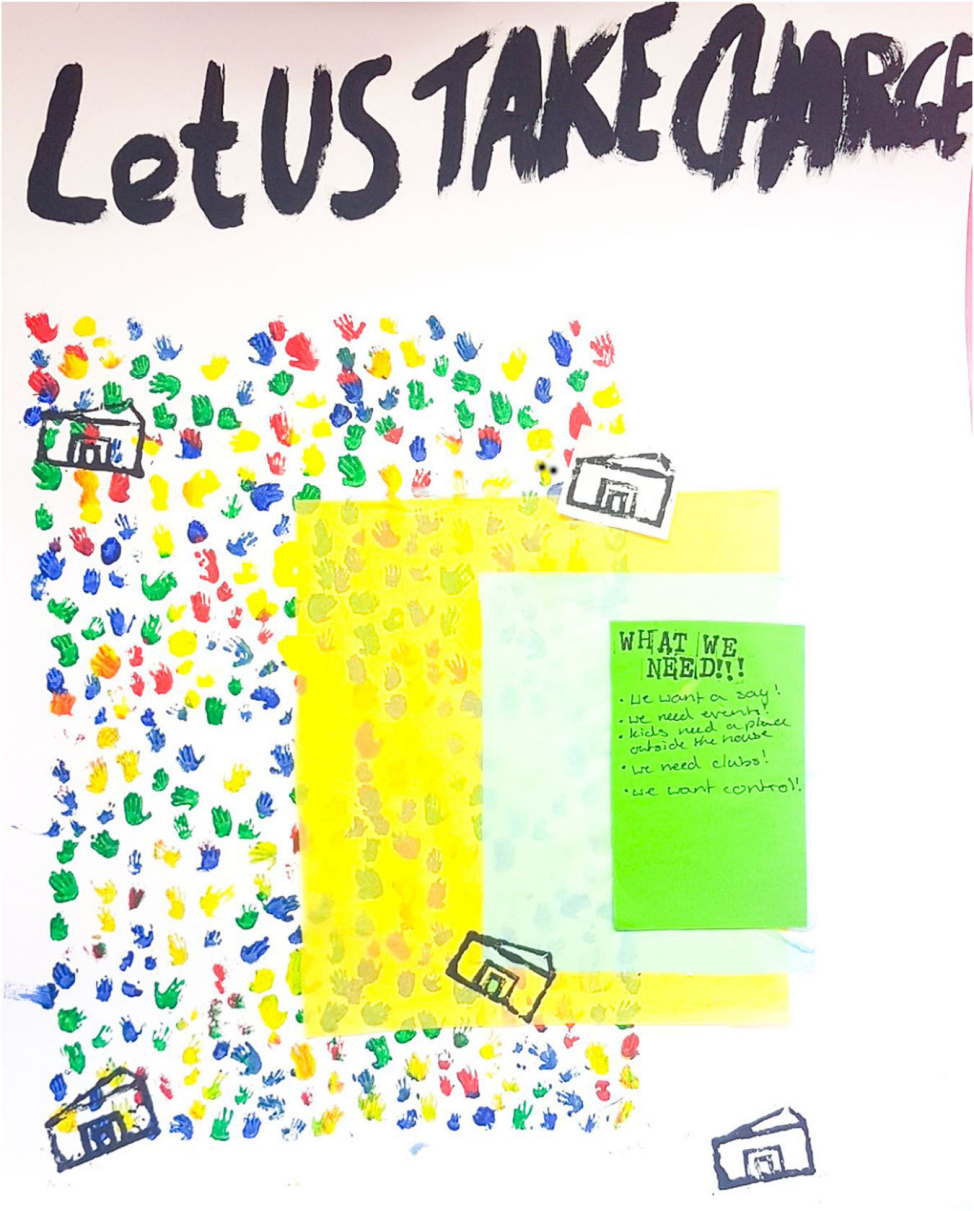
Poster entitled ‘Let US TAKE CHARGE’ featuring: a repeated print of small hands in blue, yellow, red and green and layers of rectangular yellow and blue tissue paper; green rectangle contains the words ‘What we need!!! We want a say! We need events; Kids need a place outside the house; We need clubs! We want control!’; printed image of a community building (Glasgow 2, she/her, 15).

Artwork (Figure 1) from Glasgow 2 conveys a similar preference.

The artist explained: ‘I really wanted to have a pattern in it, so using little hands would be a good idea to symbolise that communities, that are being brought together, make decisions’ (Glasgow 2, she/her, 15). Across groups, discussions often focused on moving towards systems of governance in which communities play a greater role in decision‐making. These suggestions appeared to emerge from concerns that key institutions (local, regional and national governments) and existing democratic arrangements perpetuated inequalities in power, in ways that some participants thought predicated health inequalities. This echoes population health experts emphasis on the importance of considering power as the essential element in generating or reducing health inequalities.^34^

Despite a general sense, across groups, that changes to the political system were needed (primarily ceding power to citizens and communities), these views were not universal. One participant conveyed her alternative perspective in an interview:

L3_P5: I don't think it's the right approach to take down the government because I think we need stability, I think the way it's run isn't the right way and that's where we need to change, so we shouldn't be like altering the system, we should be working with it to change it. (Leeds 3, she/her, 17)

This participant was cautious about the revolutionary rhetoric some participants in Leeds 3 were adopting, though remained committed to change. Another perspective from Glasgow 3 shifted focus from local notions of community to thinking in more global, planetary terms:

G3_P4: And for rules and stuff like that I wrote, the rules of the people […] I hope that in a utopia like far, far, far, far, far, far, far future, it's less of governments in localised places, like countries and things like that. […] But I feel like human beings will progress to the point where we're just like; countries are a bit stupid; we're going to planets now. (Glasgow 3, he/him, 18)

Visions of alternative systems of democracy, therefore, were not unified. Despite this, consensus was built around a concern to disentangle control over policy change from current systems reflecting changing patterns of political engagement amongst young people, with a preference for alternative over formal modes of political participation.^35^ Young people's voices perhaps then lend support to calls for a *democratic rejuvenation* to address health inequalities in the United Kingdom, which would involve embracing democratic innovations and participatory decision‐making to reduce those power imbalances which predicate health inequalities.^36^

#### 3.2.2 Shared and sustainable future—environment

Young people's utopian conceptualisations and policy proposals regarding the environment illustrate the wide‐reaching positive impacts they conceived improving places could have on health. When asked to envision a utopian society, initially many of the young people focused on physical aspects of the environment with a connection to nature prioritised. One participant described ‘blue skies’, ‘palm trees’ and ‘seagulls’ (Leeds 2, she/her, 18) while another listed, ‘fruit trees, plants, greenery, flowers, land, clean’ (Glasgow 2, she/her, 17). A sensory spoken word performance about Utopia also suggested this preference for living close to nature:

L2_P3: I can hear the rustling of the wind. I can hear nature. I can hear live music. I can't hear violence or shouting. I cannot hear cars or trains. (Leeds 2, he/him, 17, showcase)

Links made by young people between access to nature and mental and physical health broadly reflect evidence on the positive effects of exposure to green space on levels of health inequality within populations.^37^

**Figure 2:**
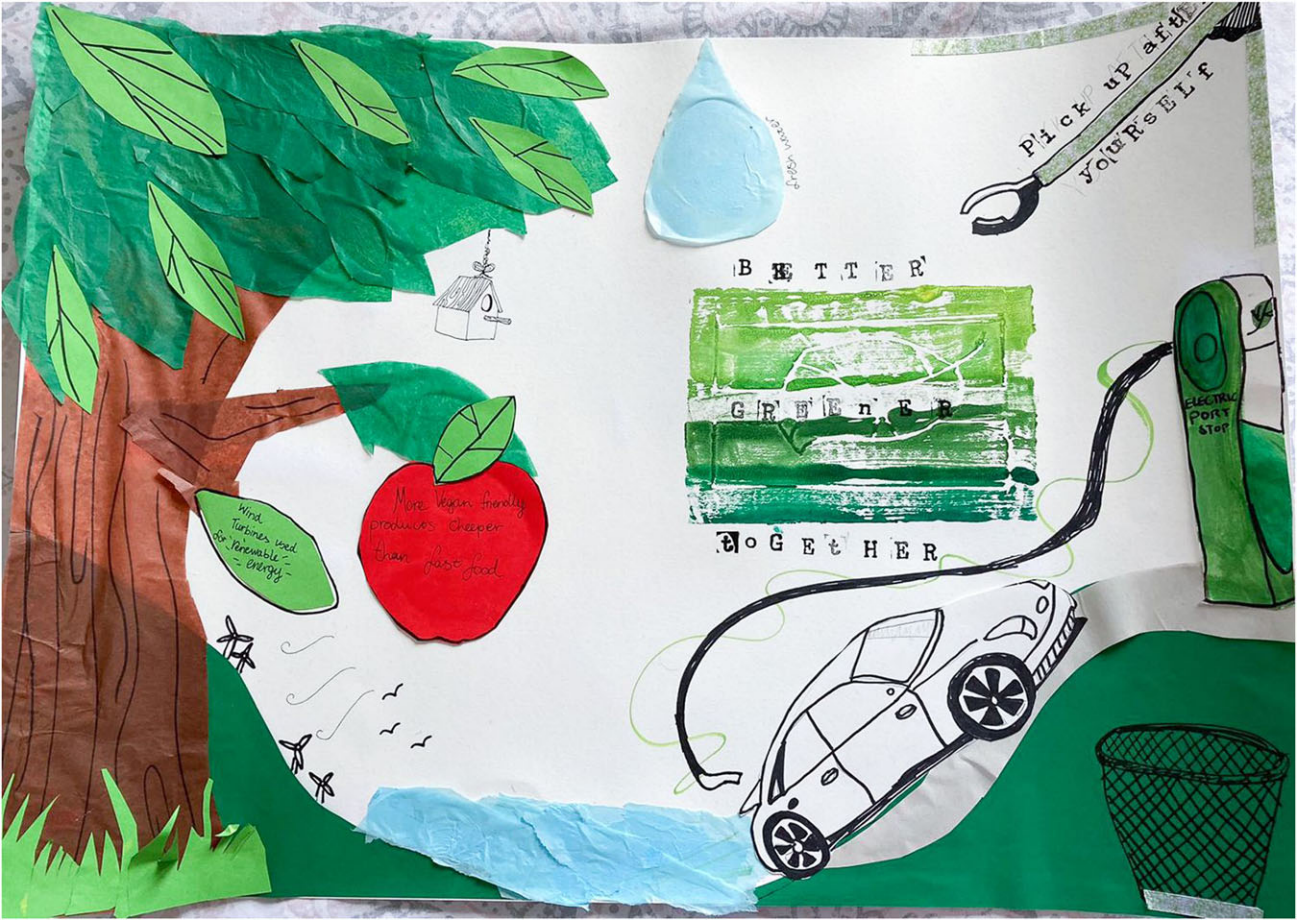
Poster collage featuring: a tree collage with text on a leaf ‘Wind turbines used for renewable energy’, an apple with text ‘More vegan friendly products cheaper than fast food’ and a bird house; a hill with wind turbines and birds flying; a body of light blue water and a large raindrop labelled ‘fresh water’; an electric car, charging station and an empty rubbish bin; a litter‐picking tool with text ‘Pick up after yourself’; printing of a leaf with text ‘BETTER, GREENER, TOGETHER’ (Glasgow 3, she/her, 19).

Several participants extended their discussions of natural environments beyond benefits to individuals or communities to focus on sustainability and planetary health. One participant made this a central theme in her final artwork (Figure 2).

She described the aim of the work: ‘I decided to make my poster about environmental alternatives and the importance of coming together to make the world a greener place’ (Glasgow 3, she/her, 19). Links to cooperation and community are evident but in combination with considering sustainability. Several young people tied together concerns for human, animal and environmental health, as interconnected and interdependent goals, in line with wider concerns for the converging crises of health and climate change.^38^

Addressing inequalities in access to green space was also discussed. While some suggested bigger individual gardens were desirable, or ‘green spaces for all flats’ (Glasgow 2, she/her, 17), many focused more on common areas, owned and enjoyed by all:

G3_P3: I think there is a lot of unused space that could be used to make it greener, like putting gardens on top of it, namely roofs, so I think there could be many benefits to this. I think it would be more space for people to hang about, it would be a fun thing to get into gardening and take care of it. (Glasgow 3, he/him, 18)

L3_P1: Like, they could have, like a lot of, like, flowers and plants, and things, so like, people can go, and like, take plants if they need it, like, grow food there. (Leeds 3, she/her, 15)

Many participants envisioned a social or community element to the green spaces they described, whether places to ‘hang about’ or grow food to share. These remarks suggest intuitive understandings of theoretical pathways through which contact with nature is expected to increase social cohesion, through fostering relationships with neighbours and increasing a sense of community, which in turn is seen as likely to improve health and wellbeing.^39^

Participants also discussed the built environment and housing. Concern to ensure a high quality of housing for all and to prevent or mitigate homelessness was discussed across several groups, reflecting well the importance of housing for health^40^ and the potential of housing interventions to reduce inequalities.^41^ This became a central theme in a group spoken word performance featuring the line: ‘We want affordable housing for everyone’ (Leeds 3, she/her, 16). Other housing‐related solutions were proposed in Leeds 1:

L1_P4: There're a lot of buildings that are like really important but they're not being used just because they're just like really important buildings. They're completely empty. Those could come in use and people could benefit from them. But how is it they've just kept them there just for people to admire instead of actually…

Facilitator: Do you mean like buildings that don't have anybody in that are like abandoned, or…?

L1_P4: Yeah.

Facilitator: Is that what you mean?

L1_P4: Those ones are like buildings that maybe the Royal family used to live in and no one uses anymore, it's like a tourist site, or…

Facilitator: Brilliant, yeah, get people in them.

L1_P1: Like the Queen has how many castles? She's got…

Facilitator: Oh gosh, so many.

L1_P1: Well she doesn't live in them all, does she?

(Leeds 1, she/her, 15; she/her, 19)

Although participants across groups did not always draw distinctions between income and wealth, interest in addressing vast inequalities in housing and property suggests some young people's concerns to address inequalities in wealth, at least in terms of property. This notion is partly aligned with wider calls for mechanisms such as land reform and inheritance tax, to reduce wealth inequalities, as potentially important ways of addressing health inequalities.^42^, ^43^

#### 3.2.3 Inclusive future—society/culture

The values of a well‐functioning society were central to young people's discussions of alternative futures, reflecting the core position of ‘cultural and societal norms and values’ in some prominent conceptual frameworks of health.^44^ Across all groups, open and supportive communities were imagined, free from discrimination and promoting empathy and respect. One participant commented on his vision:

L2_P2: I also put acceptance. I also felt free, that no one was judging me, because usually, when I'm in town, I'm, you know, all gothed up and everything, I get stares, slurs, all that good old stuff. I also felt less alert when I was there.

Facilitator: Yes, because there's no need to be alert?

L2_P2: No need to be alert. (Leeds 2, he/him, 19)

**Figure 3:**
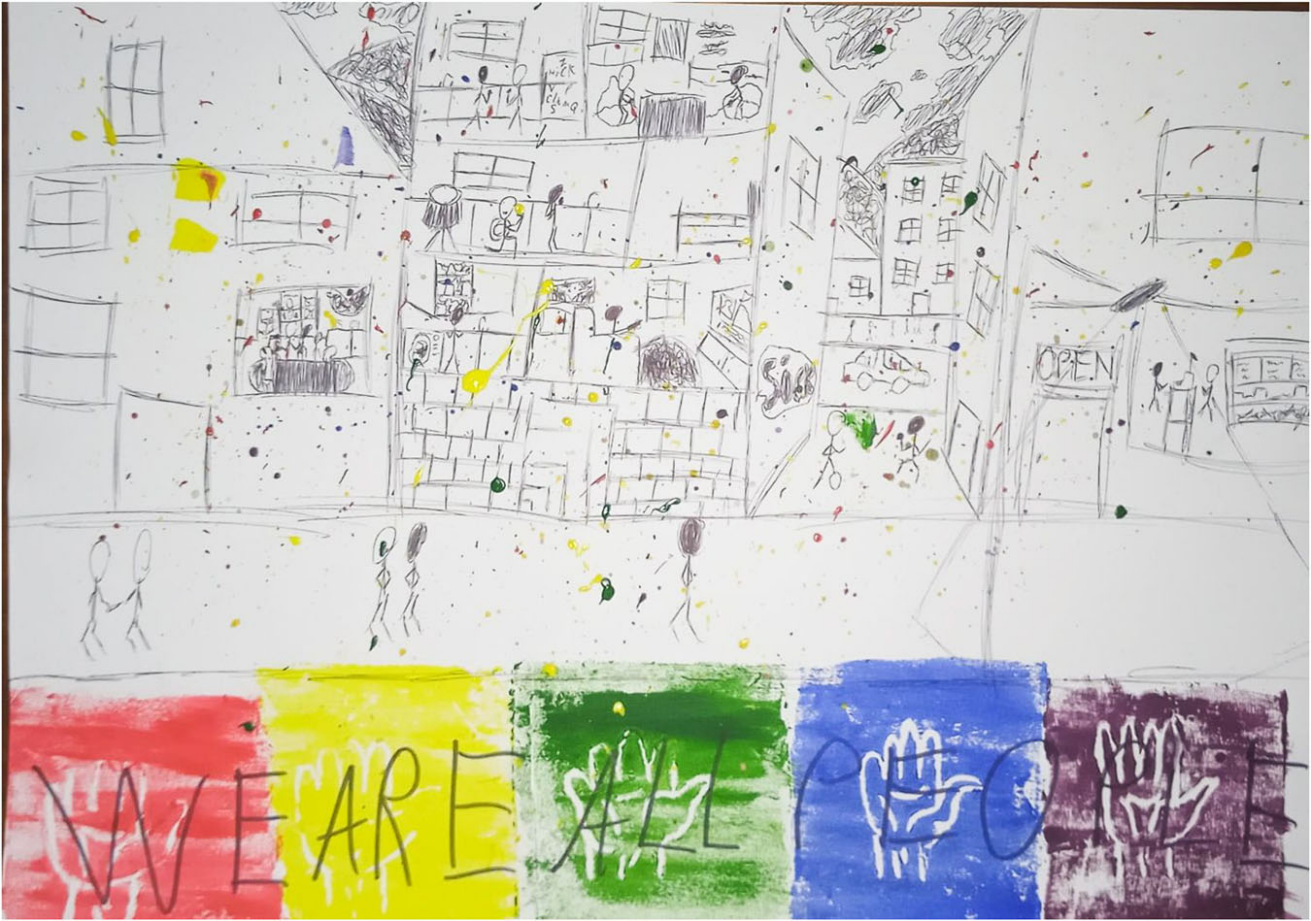
Poster featuring: line drawing a cross‐section of a street; stick figures walking and playing a game outside, and cooking, eating and standing at a bar with an ‘open’ sign inside; repeat hand print in rainbow colours with the words ‘WE ARE ALL PEOPLE’; colourful paint splatter (Glasgow 3, he/him, 18).

This participant juxtaposed acceptance in utopia to a current reality in which identities and practices are constrained by concern, even fear, about people's reactions. This aligns with existing qualitative accounts of social determinants which identify fear and stigmatisation as important factors constraining health (by restricting people's access to spaces and activities) and which suggest that such experiences are not equally distributed but concentrated in already disadvantaged communities.^45^, ^46^ Addressing similar concerns, Figure 3 depicts a harmonious society.

The artist reflected on how unity is featured in his artwork:

G3_P4: When I think of utopia, I don't think of like everything's perfect […] which is technically what a utopia should be, […] when I think of like what I would like the planet to be, I think of more of like all people together, it's like all people against the problems instead of separating each other […]. So like there's still like sadness in family and people getting caught in the rain, but it's everyone kind of together against the problems. (Glasgow 3, he/him, 18)

Population health experts consider discrimination an important and under‐researched influence on health, with various individual and structural pathways through which discrimination impacts health.^47^ Experiences of discrimination are also a common feature of lay accounts of the social determinants of health.^46^, ^48^ That diversity and inclusion were central to participants' improved versions of society supports calls for increasing social cohesion and eliminating discrimination to address inequalities.

When asked about how these visions might be achieved, some participants described schools as a venue for cultural change. For example, young people across groups supported improving curricula through consideration of the UK's role in the slave trade, racism, bullying and lesbian, gay, bisexual, transgender and questioning+ issues:

G3_P2: Well one thing that they need to put in schools earlier is more on the LGBT stuff […], when kids start to understand it because kids are just coming out and they don't really know anything about it. And then you're getting people making fun of them, ‘cause they don't understand anything about it.

[…]

G3_P1: Yeah. I included that in my social stuff as well. Like, LGBT acceptance and BLM acceptance because those are important things. (Glasgow 3: G3_P she/her, 16; G3_P1 she/her, 19)

Across groups, efforts to address injustices related to ethnicity, gender, sexual orientation and identity were well supported, reflecting a concern for considering multiple axes of inequality, as well as the institutions in which these are produced. Here, young people's assertions align with calls for intersectionality‐informed approaches to health inequalities, paying attention to the imbalance of power that underlies intersecting axes of inequality, shapes social position and influences health.^49^

Articulations of injustice related to class or differences in income and wealth were more varied. For some, discussion of class/material inequalities was less concerned with injustice and discrimination (as above) and more with sympathy and pity, and proposed solutions reflected this:

L3_P4: […] Say you're really rich and you're not fully aware to the extent of someone who's of a lower class, you're not understanding how much less money that they'd earn or get compared to you. […] I think you've either got to give them some statistics or you've got to show them […] to make them feel real sympathy and see if that will hopefully get to the heart. (Leeds 3, she/her, 16)

In contrast to this call for sympathy from the most advantaged, another participant's utopian vision comprised: ‘Unemployment not being stigmatised, but everybody would be supported rather than ostracised. Erasing the concept of the powerful and powerless’ (Leeds 3, they/them, 14, paraphrased to whiteboard). This latter framing echoes critiques of the shortcomings of dominant models of charitable social support in reinforcing power imbalances (e.g., food banks) and calls for a more rights‐based approach.^50^ Participants' discussions also considered whether individuals deserve extremely high incomes:

G1_P6: Jeff Bezos worked really hard to get to where he is. I'm not saying the person who's paid £120 hasn't worked really hard but they've not created something which is world widely used.

G1_P2: Revolutionary.

G1_P6: Revolutionary, yes.

G1_P7: But what if they didn't have the opportunity to or the qualifications?

G1_P6: You don't need qualifications to make something revolutionary.

G1_P7: Not a lot of people pay attention to you if you don't.

G1_P6: Well, I'm assuming that Jeff Bezos, people didn't listen to him at first. I don't think it's unfair.

(Glasgow 1: G1_P6 he/him, 17; G1_P2 he/him, 15; G1_P7 she/her, 17)

G1_P7: I think a lot of people that have money are just, like, putting it down the drain and just burning it basically with how they're using it. And, like, if…the common phrase keeps coming up, like, ‘eat the rich’ that's coming back into popularity in 2020. Like, the rich get richer and the poor get poorer. It's, like, they're cutting funds from needed services… (Glasgow 1, she/her, 17)

**Figure 4:**
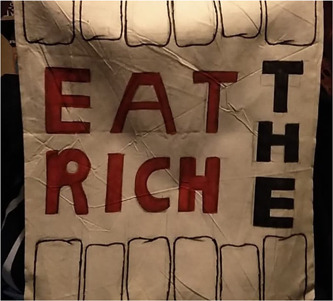
Tote bag featuring: line drawing of top and bottom teeth; ‘EAT THE RICH’ in bold black and red letters in the centre (Glasgow 1, she/her, 17).

Figure 4 and participants' discussions in Glasgow 1 reflect the multiplicity of views on material inequality. For some, hard work or entrepreneurial enterprises may justify enormous wealth while, for others, excess wealth is unjust. Similar disagreements on justifiable levels of income inequality feature in previous research on public perspectives on addressing health inequalities.^10^ While young people's visions of accepting and united futures broadly aimed to foster equality and tackle discrimination, addressing material inequality was more contested, perhaps related to concerns about stifling aspiration, which has been described as a central motif of contemporary society for young people.^51^

#### 3.2.4 Generous and balanced future—economic

Young people's visions of economic futures broadly focused on three main areas: employment and the labour market, pay and welfare/universal basic income (UBI), with concerns around addressing injustices and protecting people's mental health evident across each. In general, the visions young people shared of employment focused on wellbeing. One participant wrote of her ideal model of employment: ‘People work half the week and rest the other half’ (Glasgow 2, she/her, 14). Similarly, in Leeds 3, one participant contributed: ‘There would also be paid hobbies with hourly rates. Choose how much you work. […] Encourage work to benefit the community, i.e. picking up rubbish’ (Leeds 3, she/her, 16, paraphrased to whiteboard). For many of the young people, work was reconceptualised as protecting the health of individuals and communities in terms of what it entailed (enjoyable activity), what it contributed to (communities, not profits) and how much was required (enough, not more). These suggestions echo calls for the kind of substantial policy shift that some researchers and advocacy organisations promote in which wellbeing (rather than traditional measures of economic productivity) is placed at the heart of policymaking.^52^ However, participants also recognised that few jobs in contemporary society meet the criteria described, and some returned focus to tangible employment difficulties (pandemic‐related employment challenges featured prominently). Links were frequently discussed between employment and mental health, in ways that align with reviews of research evidence^53^ around employment as a driver of mental wellbeing: ‘Yeah, a lot of people are anxious, and because they lose their job and things, so it does cause anxiety and pressure on a person’ (Leeds 3, she/her, 16). While prioritising work‐life balance and enjoyable work in future visions, income stability and job security were pressing contemporary policy priorities.

Discussions across all groups included proposals to address levels of pay, with an emphasis on fairness. Some interpreted fairness as equal pay for equal work, including addressing the disparity in UK minimum wage by age (from April 2023 UK National Minimum/Living Wage ranged from £5.28 [under 18] to £10.42 [23 and over]^54^). Others suggested key workers (especially healthcare and retail staff) should be paid more, echoing wider public and media sentiments, which reframed jobs previously considered ‘low‐skilled’ with new prestige during the pandemic.^55^ Some advocated for equal pay for everyone in a commitment to absolute equality, while others struggled to envisage a world without inequality:

Facilitator 1: …[L2_P1] said that everyone should be paid the same – what do people feel about that?

L2_P2: I'm pretty sure she means by everyone as in it doesn't matter what your race, gender is, I'm pretty sure that's what she means. Not by, oh yeah, doesn't matter what job you do, you still get paid the same. I'm pretty sure she doesn't mean it like the latter. But yeah, it sounds pretty cool.

Facilitator 2: You're shaking your head. What did you mean by that, do you want to explain?

L2_P1: I did mean everyone get paid equally, like no matter what gender and stuff, but also for work and things. Like depending on the job, maybe some people should get paid a bit higher, depending on what it is. But I feel like everyone should get equal pay, but not like a low amount, like a higher amount personally. Because then everyone could just afford the same things. You wouldn't have people looking down on others and stuff like that, and it would just be more equal. And less people would be struggling with money […] when really everyone needs the same amount of money to have a really good life. (Leeds 2: L2_P2 he/him, 19; L2_P1 she/her, 20)

Resisting the concept that certain jobs, skills or people are valued more highly than others via income differentials, this participant expressed her preference for incomes reflecting need. The result, as framed by the participant, would be a reduction in the negative consequences of inequality (such as status anxiety), echoing claims about the pernicious effects of inequality.^56^ Distributing material resources according to requirements for living well (rather than via systems seen to perpetuate inequality) garnered support in most groups. Although there was no consensus about how or whether wages should be equalised, there was general agreement that wages for the lowest paid, particularly key workers, and young people, are currently inadequate in the United Kingdom.

Participants also discussed overhauling the UK welfare system to better address poverty, a goal that was universally supported. Interest was expressed by most groups in UBI, an unconditional income for all, which some experts argue has the potential to address health inequalities globally.^57^ Ensuring basic needs were met was prioritised:

L3_P4: Yeah, I think because you've got to have basic necessity, but what you've got to have, it's heating, food, and water, I believe, or electricity, or something.

Facilitator: Yeah.

L3_P4: And I feel, saying to people, look, you've got to have that as basic necessity, you've got to have it, but yet, they're not providing for it. So, I feel like, if they have a basic income to provide for at least that, I feel like it would be more beneficial to everyone. Because, they can't say, oh well you've got to have it, but not provide it, because it's just wrong. (Leeds 3, she/her, 16)

Despite the emphasis on the importance of community‐level governance, widespread support for improving safety nets, aligned with recent calls from researchers,^36^ suggests participants valued national‐level policy action on this issue. Across employment conditions, pay and welfare arrangements, young people's directions for action prioritised wellbeing over economic growth, echoing interest in transformative approaches to policymaking postpandemic.^58^

## 4 CONCLUDING DISCUSSION

Taken together, these visions demonstrate diversity in participants' views about improving governance, environments, society/culture and the economy, but also a high degree of consensus around a need for systemic changes to achieve reductions in social inequalities (and the health differences that flow from these). This broadly aligns with recent research in deprived areas of England which found that young people have a good understanding of the ‘upstream’ drivers of health inequalities.^12^ Preferred solutions also echo broader public perspectives, with young people in our study consistently emphasising the need to improve living and working conditions, echoing the findings of citizens' juries with adults in Glasgow, Liverpool and Manchester.^10^ Likewise, our participants called for structural, long‐term solutions (and not individual‐level behavioural interventions) aligning with participants in a Q‐methodology study of remote and rural communities.^9^ The young participants in our study also broadly agreed on the importance of addressing poverty (e.g., through increasing wages and improving safety nets) with considerable (though more contested) support for reducing income and wealth inequalities. Some participants supported greater consideration of the mechanisms through which economic resources flow, particularly in relation to wealth accumulation (e.g., property), consistent with recent calls from public health researchers.^43^

Throughout the project, the extent to which young people were focused explicitly on health varied. At times, links to health were clear, particularly with reference to how some determinants and solutions might impact mental health. Framings of environmental solutions (contact with greenspace) or social/cultural solutions (addressing discrimination) included pathways to health impacts. When discussing proposals for more participatory governance and community decision‐making, however, the specific pathways via which health might be influenced were often not articulated explicitly, though the perceived need to address unequal power relations was consistent across discussions. This aligns with existing research; while inequalities in power have been identified as a fundamental cause of health inequalities,^34^ and the World Health Organisation has called for work to strengthen community engagement in governance systems,^59^ research examining the pathways connecting participation in policy‐making to health outcomes remains at a nascent stage.^60^

Other studies employing the concept of utopia to explore inequalities have consciously focused on envisaging futures with young people that are not explicitly linked to health, to free up discussions and enable participants to prioritise those issues most important to their current experiences.^61^ In our study, facilitating creative discussions around participants' utopian visions as a separate exercise, following earlier group reflections on the drivers of health inequalities, appeared to function similarly, but participants' concerns to address social inequalities, including those related to power and decision‐making, also appeared to reflect broad ‘upstream’ understandings of the determinants of health and health inequalities. Future studies exploring public perspectives on addressing health inequalities, including those drawing on notions of utopia, might reflect further on whether ideas and priorities change depending on the extent to which health is explicitly prioritised since this may have implications for both our understanding of public views and for thinking about possible alternative framings of public and policy conversations about reducing health inequalities.^62^

As previously noted, variations in existing research suggest that the specific questions and approaches taken to exploring public perspectives on addressing health inequalities influence the accounts generated. Politically innovative proposals suggested by our young participants, including increasing participatory governance, are notably absent from existing studies,^8^, ^9^, ^10^ although these ideas have recently been emphasised by researchers.^34^, ^36^ That young people developed these proposals and positioned them as key to reducing social inequalities via reducing inequalities in power relations as well as through enabling policy choices that are likely to reduce inequalities, seems a testament to both the sophistication of young people's insights and the potential of engaging with notions of utopia^23^ in conversations about tackling health inequalities.^24^ Rather than generating discussions bound by concerns about feasibility, visionary proposals, that could be characterised as both unrealistic (in terms of the level of change required) and realistic (in how likely they might be to generate reductions in health inequalities), is perhaps helpful in moving forward debates on actions to address health inequalities.

In terms of limitations, our study engaged groups of young people in discussions and creative activities focused on addressing health inequalities; however, some perspectives were likely not well‐represented. Limited information was collected on participant sociodemographics. More detail may have allowed us to explore how individual and social identities influenced perspectives. The study was also limited in terms of content covered. A longer‐term project, conducted over several weeks (meeting less frequently), would have allowed for engagement with evidence and discussion of the range of social determinants and axes of inequality that characterise understandings of population health inequalities and for the responsive pursuit of evidence most relevant to emerging priorities. However, the young people were variously committed to other activities and may have been unable to commit to a longer‐term project. Further, while art‐making and creative engagement were prioritised within the project, detailed exploration of artworks and performance pieces during the process of data analysis was limited. The inclusion of visual and performance artworks was generally limited to triangulation with textual data rather than standalone analysis.

Despite these limitations, our research with young people suggests that calls for wider public conversations around the social determinants of health^3^, ^4^ are likely to be met with both willing participants and wide‐ranging reflections on the range of actions needed to address health inequalities in the United Kingdom. Proposals for systemic change to achieve reductions in social inequalities and the health differences that flow from these are in many ways well‐aligned with those expressed by population health researchers and advocates.^58^ Researchers could also do more to collaborate with citizens and communities on research and advocacy efforts, to influence policy decision‐making around the kinds of ‘upstream’ policies that both young people and population health researchers support.

## AUTHOR CONTRIBUTIONS

Gillian Fergie conceived and designed the research project with expert input from Katherine Smith, Mhairi Mackenzie and Shona Hilton. Gillian Fergie and Thu Thuy Phan jointly designed (with creative organisations) participant workshops and coordinated these, including all data collection. Caroline Vaczy and Gillian Fergie coded and analysed the data with input from Katherine Smith, Mhairi Mackenzie and Shona Hilton. Gillian Fergie, Caroline Vaczy and Katherine Smith conceived the paper and co‐authored the first draft. All authors contributed to drafting and revising the paper and approved the final version.

## CONFLICT OF INTEREST STATEMENT

The authors declare no conflict of interest.

## Data Availability

The data generated as part of the wider study are available to registered users with access to safeguarded data through the UK Data Service as Fergie, Gillian M. (2022). Creative Insights: Developing a Participatory Approach for Exploring Young People's Perspectives on Health Inequalities, 2019–2022. (Data Collection). Colchester, Essex: UK Data Service. 10.5255/UKDA‐SN‐855952.
